# Mix of methods is needed to identify adverse events in general practice: A prospective observational study

**DOI:** 10.1186/1471-2296-9-35

**Published:** 2008-06-15

**Authors:** Raymond Wetzels, René Wolters, Chris van Weel, Michel Wensing

**Affiliations:** 1 Radboud University Nijmegen Medical Centre, Centre for Quality of Care Research, P.O. Box 9101, 6500 HB Nijmegen, The Netherlands; 2 Radboud University Nijmegen Medical Centre, Department of General Practice, P.O. Box 9101, 6500 HB Nijmegen, The Netherlands

## Abstract

**Background:**

The validity and usefulness of incident reporting and other methods for identifying adverse events remains unclear. This study aimed to compare five methods in general practice.

**Methods:**

In a prospective observational study, with five general practitioners, five methods were applied and compared. The five methods were physician reported adverse events, pharmacist reported adverse events, patients' experiences of adverse events, assessment of a random sample of medical records, and assessment of all deceased patients.

**Results:**

A total of 68 events were identified using these methods. The patient survey accounted for the highest number of events and the pharmacist reports for the lowest number. No overlap between the methods was detected. The patient survey accounted for the highest number of events and the pharmacist reports for the lowest number.

**Conclusion:**

A mix of methods is needed to identify adverse events in general practice.

## Background

Patient safety is important in primary care, as most patients and most of their health problems are treated in this setting [1]. Adverse events in primary care occur between five and 80 times per 100 000 consultations [2]. General practitioners (GPs) were positive about reporting adverse events [3], but the validity and usefulness of incident reporting systems remains unclear [[4], page 3]. A range of methods is available for identifying adverse events, such as review of medical records and case reviews of deceased patients [5-7]. The aim of the presented study was to compare five different methods for identifying adverse events in general practice with respect to the number and type of identified events, the patient subgroups affected, and the agreement of events across the methods.

## Methods

### Study design and setting

A prospective observational study was performed focused on five GPs in two practices in a period of five months (May to October 2006). A total of approximately 8250 patients were registered with the two practices. The ethical committee of the Radboud University Nijmegen Medical Centre approved the study.

### Measures

We defined an adverse event as an unintentional event with actual or potential harm to the patients' health status. This broad definition is consistent with the International Patient Safety Event Classification of the World Health Organization: "a process or act of omission or commission that resulted in hazardous health care conditions and/or unintended harm to the patient" [8]. The five methods were: GPs reported adverse events, pharmacist reported adverse events, patients' experiences of adverse events, assessment of a random sample of medical records, and assessment of all deceased patients.

#### GP reported adverse events

The GPs recorded all events during a five-month period. On the basis of an existing international taxonomy for errors in general practice [9] a simplified computerised registration form was made. The GPs registered event date, birth date of patient, gender, event category (practice administration (archive; medical record; appointment; other), diagnostic (wrong diagnosis; delayed diagnosis; missed diagnosis; other), therapeutic (wrong, incomplete; delayed; none, though it should be; other), communication (with patients; with caregivers; other)), and additional remarks and/or context.

#### Pharmacist reported adverse events

The pharmacist recorded events from her point of view in the same period. An adjusted form was developed for this purpose. Event date, birth date of patient, gender, practice, event category (prescribing error (wrong prescription, wrong administration; wrong dose; other), adverse reaction (adverse reaction; allergic reaction; overdose; interaction; contra-indication; other), dispensing error (too late; wrong medicine; wrong dose; other)), and additional remarks or context were recorded.

#### Patient reported adverse events

In the waiting room of the two practices samples of 50 patients, consecutively visiting the practice, were invited to complete a questionnaire on experienced problems with safety of their health care in the previous six months. A drop box was used to collect the completed questionnaires. Questions were derived from items of the Medical Harvard Study, [10] and from questions of two survey studies. [11,12] Questions had to be answered with yes or no; and asked whether something had gone wrong in the care they had received from their GP during the past half year (see table 1 for the complete questionnaire). The questionnaire guaranteed anonymity of participating patients.

**Table 1:** Feasibility, yield, usefulness and reliability per method

Method	Feasibility	Yield	Usefulness	Reliability*
General Practitioners registering events	+/-	+/-	- Different kind of events - Setting areas for improvement per practice in patient safety	-
Pharmacist registering events	+	-	- Only medication errors - Guidance in improving patient safety in prescribing medication	-
Patients' questionnaire about patient safety	++	+	- Different kind of events - Revealing GPs' blind spots - Setting areas for improvement per practice in patient safety	+/-
Random audit of medical records	+/-	+	- Mostly therapeutic and communication events - Time consuming	+
Audit of medical records of deceased patients	+	+/-	- Different kind of events - Low number of patients	-

*Is this measure a reliable estimate for the actual number of events in general practice

#### Assessment of medical records

Thirty medical records per GP (in total 150 medical records) were randomly selected (using a list of random numbers) from patients who had visited their GP in the observation period. Anonymous medical records, containing the information from this period were printed out. Two clinical researchers examined the information independently [RWo, RWe]. They scrutinised the records for indications of events and, when found, categorised the event (errors in office administration, diagnosis, treatment or communication with their subcategories); and added demographic data of the patient (birth date, gender). Subsequently the GPs discussed their findings and reached consensus.

#### Assessment of all deceased patients

Both practices had a registration of deceased patients from which the medical records were retrieved of all patients who had died in the period May-October 2006. One GP examined the medical records of these patients for events. The same registration form and analysis procedure as for the audits of medical records was used.

### Data-analysis

Numbers of events per category per method and overall were added up and percentages were calculated. Age range, mean age, age category (<50 and > = 50 years old) and percentage of women were calculated per method and overall.

A qualitative analysis of events will be published elsewhere. [13]

## Results

Table 2 presents the events reported by the five methods. A total of 68 events were identified using these methods. Each of the methods provided events that were not found with other methods; no overlap between the methods was detected. Mostly women were affected, in almost two-third of the registered events. Patients over 50 years and patients under 50 year were almost equally affected, but the age group below 18 was rarely involved. An exception to this general trend was the subgroup of events based patient registration, which involved mostly patients under 50 years of age.

**Table 2:** Overview of events, characteristics of patients involved and event categories

	Total number examined	Number of events	Mean age	Age range	Age <50 yrs	Age >50 yrs	% Female	Events in office administration	Events in diagnosis	Treatment events	Events in communication
GP registration	20	20	57	5–91	30% (6/20)	70% (14/20)	70% (14/20)	35% (7/20)	25% (5/20)	15% (3/20)	25% (5/20)
Pharmacist registration	6	6	62	40–90	33,3% (2/6)	50% (3/6)	66,7% (4/6)	-	-	100% (6/6)	-
Patient registration	91	27	44	17–75	69,2% (18/26)	30,8% (8/26)	66,7% (18/27)				
Random records	150	11	57	34–75	36,4% (4/11)	63,6% (7/11)	36,4% (4/11)	18,2% (2/11)	9,1% (1/11)	36,4% (4/11)	36,4% (4/11)
Deceased records	28	4	75	33–94	25% (1/4)	75% (3/4)	100% (4/4)	25% (1/4)	25% (1/4)	-	50% (2/4)
Total	295	68			47% (31/66)	53% (35/66)	64,7% (44/68)				

### GP reported events

GPs registered 20 events in five months; in these months there were 4095 patients who had visited the practice, resulting in almost 5 events per 1000 patients visiting the practice. Most of these events concerned women aged 50 years or older. GPs registered little events that concerned treatment.

### Pharmacist reported events

The pharmacists registered six events per 16320 prescriptions (including repeated prescriptions), all related to female patients. Of these there were five prescribing errors and one known allergic reaction.

### Patient reported events

Twenty-seven patients answered positive to one or more items of the patient questionnaire. In total, 78 positive answers were given, it is unclear whether these answers referred to one or more events. Patients answering positive to questions were slightly younger compared to all responders (43,8 vs. 45,5 years old). By far most often (16/78) mentioned was a breach of confidentially of their medical record during treatment by their GP. Next, a lack of respect by GP or practice assistant was reported (8/78), followed by a delay in diagnosis (5/78), inappropriate drug prescribed (5/78) or inappropriate advice given (5/78) and a wrong appointment at the practice (5/78).

### Assessment of medical records

There had been 4,095 patients consulting the practices during the study period, from which 150 medical records were randomly selected. Analysis resulted in the finding of 11 events, all errors of treatment and communication.

### Assessment of deceased patients

During the study period 28 patients had died. One medical record was not available for examination, as this patient had not given permission. This method generated between one or two events per ten deceased patients.

## Discussion

All five methods proved to identify a number of adverse events. The patient survey accounted for the highest number of events and the pharmacist reports for the lowest number. All methods resulted in a variety of events, except for the pharmacist reports, which only referred to pharmaceutical treatment. The identified events referred to adult male and female patients of all ages, but events on children were very seldom reported. Events based on patient registration mostly involved individuals aged 50 years or younger. There was no overlap between the methods regarding the identified events.

A systematic review of methods to identify adverse events in health care concluded that "the available methods have widely differing purposes, strengths and weaknesses and must be considered as complementing each other by providing different levels of qualitative and quantitave information" [[4], page 4]. Incident reporting systems have the advantage of being not as time-consuming as formal studies, but they are likely to underestimate the number of adverse events (numerator) while the number of opportunities for incidents (denominator) remains unknown. A recent study in hospitals also concluded that different methods should be used in order to provide an adequate assessment of clinical adverse events [14].

Our study in general practice suggested that GPs did not report all adverse events, as compared to other sources. It was difficult to assess the validity of the GP reported events because of the lack of overlap with other methods. Event reporting by GPs is probably important for raising awareness and a safety culture, but it is unlikely to be a comprehensive method for identifying adverse events. None of the GPs had a feeling of suspicion with registration. They did not think the registered events would be used against them. They all felt it was difficult to remember to register events due to daily routine working procedures and time pressure.

Almost three in ten patients reported on health safety issues, and patients as a group reported a substantial number of adverse events. It was unclear whether the checked items of the patient questionnaire referred to one or more health safety issues; we assumed these were from 27 events (thus one event per patient), in order not to overestimate number of events. In this study almost 60% of patient reported events concerned psychological harm or harm in trust or confidence. Another study showed that less than a third of reported harm was physical [15]. Patients registering events may reveal blind spots in care provided by the GP. The questionnaire seemed quite easy to complete and its analysis asked for a relatively low time investment. However, the validity and usefulness of patient reported incidents needs further research [16,17].

Inaccuracies of medical record are well documented [17]. This method generated different types of adverse events, although the number in diagnostic events was rather low. It took a large time-investment to go through the medical records.

The reporting of adverse events by a pharmacist generated relatively few events. A study showed that community pharmacists correct 1% of the prescriptions [19], which obviously influences the number of remaining events. The low numbers may explained by the computerised systems, which including monitoring for potential contra-indications and interaction of drugs.

The audit of medical records of deceased patients was feasible, but the number of identified adverse events was low, and therefore its yield was restricted.

The limitations of this study should be recognized. The number and type of adverse events seemed to depend on the individual who registered. One could imagine asking other (para)medical caregivers (physiotherapists, midwifes, etc) to register as well. Also, in the communication with hospitals and referrals to specialists, things may go wrong. Specialists could therefore be a registration source of adverse events for GPs and this probably goes the other way around as well. Future studies should take this into account. Registration of events stays difficult as one kept in doubt whether an event is an adverse event or something that is generally accepted as inherent to GP care. This might have negatively influenced the number of registered events. Patients may also experience not every incident in their care as an adverse event. Whether this was the case may be influenced by the response of the doctor towards the event [20].

Patient safety has attracted "a level of public interest that the rest of the quality-improvement field in health care has failed to excite" [21]. This may be explained by a fascination for the accidental death, despite the prolonged uncertainty about the "true" estimates of mortality resulting from medical error [21]. In our study, none of the identified adverse events was associated with mortality.

## Conclusion

The public interest for patient safety has led to a number of potentially valuable initiatives to identify and prevent adverse events. None of the methods to identify adverse events proved to be superior. We suggest that more research is needed on the various approaches in general practice before their wide scale implementation can be recommended.

## Competing interests

The authors declare that they have no competing interests.

## Authors' contributions

RWe performed the study according to protocol and wrote the manuscript. RWe and RWo collected and analysed data. MW and CvW initiated and supervised the study, and developed the study protocol. All authors read and approved the final version of the manuscript.

## Pre-publication history

The pre-publication history for this paper can be accessed here:


